# TLR-4 and VEGF Polymorphisms in Chronic Periaortitis

**DOI:** 10.1371/journal.pone.0062330

**Published:** 2013-05-14

**Authors:** Fabiola Atzeni, Luigi Boiardi, Augusto Vaglio, Davide Nicoli, Enrico Farnetti, Alessandra Palmisano, Nicolò Pipitone, Davide Martorana, Gabriella Moroni, Selena Longhi, Francesco Bonatti, Carlo Buzio, Carlo Salvarani

**Affiliations:** 1 Rheumatology Unit, L. Sacco University Hospital of Milan, Milan, Italy; 2 Rheumatology Unit, Department of Internal Medicine, Azienda Ospedaliera ASMN, Istituto di Ricovero e Cura a Carattere Scientifico, Reggio Emilia, Italy; 3 Unit of Nephrology, University Hospital of Parma, Parma, Italy; 4 Laboratory of Molecular Biology, Arcispedale S Maria Nuova, Istituto di Ricovero e Cura a Carattere Scientifico, Reggio Emilia, Italy; 5 Unit of Molecular Genetics, University Hospital of Parma, Parma, Italy; 6 Nephrology Unit, Istituto di Ricovero e Cura a Carattere Scientifico Policlinico Hospital, Policlinico Hospital, Milan, Italy; University of Leicester, United Kingdom

## Abstract

**Objective:**

Chronic periaortitis (CP) is a rare disease that is characterised by fibro-inflammatory tissue surrounding the abdominal aorta and has both non-aneurysmal (idiopathic retroperitoneal fibrosis [IRF]) and aneurysmal forms (inflammatory abdominal aortic aneurysm [IAAA]). We investigated whether toll-like receptor 4 (TLR-4) and vascular endothelial growth factor (VEGF) polymorphisms were associated with susceptibility to, and the clinical features of CP.

**Methods:**

One hundred and two CP patients and 200 healthy controls were molecularly genotyped for TLR-4 gene polymorphism (+896 A/G) (rs4986790), VEGF mutations +936 C/T (rs3025039) and −634 C/G (rs2010963), and an 18 base pair (bp) insertion/deletion (I/D) polymorphism at −2549 of the VEGF promoter region. The patients were grouped on the basis of the type of CP (IRF or IAAA), and the presence or absence of established atherosclerotic disease (ischemic heart disease, cerebrovascular disease, and peripheral arterial disease).

**Results:**

There were no significant differences in the distribution of the studied polymorphisms between the patients and controls. However, carriage of the +936 T allele was significantly more frequent in the patients with IRF than in those with IAAA (26.5% *vs* 5.3%; p = 0.046; OR 6.49 [95% CI 0.82–51.54]). There were significantly more carriers of the I allele among the patients with ureteral obstruction (83.8% *vs* 58.8%; p = 0.006; OR 3.63 [95% CI 1.42–9.28]) and those who received conservative treatment (48.5% *vs* 23.5%; p = 0.015; OR 3.06 [95% CI 1.22–7.721]) than among those without, and II homozygosity was significantly more frequent in the patients with deep vein thrombosis than in those without (30.4% *vs* 11.7%, p = 0.031; OR 3.31 [95% CI 1.07–10.21]).

**Conclusion:**

The VEGF +936 C/T polymorphism may be associated with an increased risk of developing the non-aneurysmal IRF form of CP. Carriers of the I allele and II homozygosity are respectively at increased risk of developing ureteral obstruction and deep vein thrombosis.

## Introduction

Chronic periaortitis (CP) is an immune-mediated disease characterised by a fibro-inflammatory reaction arising from the adventitia of the abdominal aorta and common iliac arteries that may spread into the retro-peritoneum [1], [2]. It has non-aneurysmal and aneurysmal forms, which are traditionally and respectively known as idiopathic retroperitoneal fibrosis (IRF) and inflammatory abdominal aortic aneurysms (IAAA). As these conditions have common clinical and histopathological findings, they are considered to be the opposite ends of the same disease spectrum [1].

The pathogenesis of CP is still unclear. Earlier studies suggested that it might arise as an exaggerated localised inflammatory reaction to severe atherosclerosis plaques in the abdominal aorta. However, the absence of advanced atherosclerosis in some patients and, more importantly, the presence of constitutional symptoms, autoantibodies, and markedly increased acute-phase reactant levels, together with the frequent association with other autoimmune conditions, suggest a systemic immune-mediated process rather than a local reaction [1]–[3]. This hypothesis is further supported by the association between CP and HLA-DRB1*03, an allele linked to various immune-mediated diseases [4], and between the CC chemokine receptor 5 (CCR5) Δ32 polymorphism and the aneurysmal form of CP in patients without atherosclerotic disease [5], as well as by the marked adventitial lymphoplasmacytic infiltration of the aorta that is typical of CP [6]–[8].

Studies of aortic biopsy specimens taken from in CP patients have revealed the expression of gene transcripts consistent with lymphocytic activation (interferon-γ, interleukin(IL)-1α, IL-2 and IL-4), which supports the concept that CP may originate as an active inflammatory condition of the aortic wall [9].

Toll-like receptor 4 (TLR-4) is an important pathogen recognition receptor that plays a major role in innate and adaptive immune responses by binding to pathogens, microbial toxins, or endogen ligands such as heat-shock proteins, modified lipids and others [10], [11].

It has been shown that the engagement of TLRs induces T cell activation, as seen in immune-mediated disorders [11]. As TLR-4 and TLR-5 ligands trigger vascular dendritic cells (DCs) and induce T cell recruitment and activation in patients with large vessel vasculitides [12], TLR-4 may also play a key role in the pathogenesis of CP, which is characterised by inflammatory involvement of the abdominal aorta and the origin of the epi-aortic arteries, as well as of other large arteries such as the gastrointestinal vessels [1], [2], [13]. Recently, two co-segregating single nucleotide polymorphisms (SNPs) within the gene encoding TLR-4 (Asp299Gly and Thr399Ile) have been characterised [14] and studied in various inflammatory and infectious conditions [15]–[27], and it is known that they impair the efficacy of lipopolysaccharide (LPS)-induced signalling and its capacity to elicit inflammation [14].

Vascular endothelial growth factor (VEGF) may also be involved in the pathophysiology of CP because it is not only a potent angiogenic factor, but also acts as a pro-inflammatory cytokine by increasing endothelial permeability and inducing the chemokines and adhesion molecules that bind leukocytes to endothelial cells [28]. As a wide range of adhesion molecules have been detected in the aortic adventitia of patients with CP [29], VEGF seems to be an attractive candidate as a CP susceptibility gene. VEGF production may be partially subject to genetic control. It has been found that two *VEGF* gene mutations, +936 C/T in the 3′- UTR [30] and −634 C/G occurring in 5′-UTR (erroneously described as a +405 polymorphism in a previous study) [31], regulate plasma VEGF levels, and an 18 base pair (bp) insertion/deletion (I/D) polymorphism at position −2549 of the *VEGF* promoter region has also been described [32]. There is evidence that these three functional polymorphisms are associated with immune-mediated conditions, particularly vasculitides [33]–[38].

In the light of the above, the aim of this study was to investigate the potential associations of the two *TLR-4* and three *VEGF* polymorphisms with CP susceptibility and disease expression. In addition, we explored whether the presence of an established atherosclerotic disease influences the association between the polymorphisms and CP.

## Materials and Methods

### Study population

The study involved 102 CP patients consecutively recruited at two Italian referral centres (Milan and Parma) over a period of ten years. The diagnosis of CP was confirmed by means of computed tomography (CT) or magnetic resonance imaging (MRI) showing a homogeneously attenuated retroperitoneal mass of soft tissue density surrounding the abdominal aorta and/or the iliac arteries, without aortic displacement or bone destruction. None of the patients had any clinical or laboratory evidence of chronic infections, tumours or trauma, or a history of major abdominal surgery and/or radiotherapy, and none of them was taking any medication known to induce CP.

The control group consisted of 200 unrelated, healthy, age- and gender-matched blood donors (60% males) with a mean age of 57±13 years.

All of the study subjects were Caucasians who had been resident in Italy for at least one generation, and there were no ethnic differences between the patients and controls. The study was approved by the Ethic Committees “Provinciale of Reggio Emilia”, and written informed consent was obtained from the patients and controls before study entry.

All of the medical records of the patients were reviewed, and the data of all of the confirmed cases were recorded using a standardised data collection form that included clinical symptoms at presentation and during follow-up, co-morbidities, laboratory investigations, detailed radiological findings (CT, MRI, positron emission tomography [PET] scans, and conventional angiograms), pathological specimens (retroperitoneal biopsy), treatment regimens, response to treatment, the number of relapses, surgical interventions, and causes of death.

If baseline creatinine (Cr) levels were available, acute renal failure was defined as a rapid increase of >50% or >1 mg/dL in serum Cr levels in patients with known renal insufficiency; otherwise, it was defined as an impaired renal function (serum Cr >1.4 mg/dl) that proved to be reversible after the obstructive uropathy had been corrected (e.g. by means of ureteral stents).

Information concerning established ischemic heart disease, cerebrovascular accidents and peripheral arterial disease (PAD) preceding the onset of CP was obtained from in- and outpatient medical records, as well as by means of patient interviews whenever required. Ischemic heart disease was diagnosed if the patient had a history of acute myocardial infarction (AMI), silent AMI, revascularisation procedures, or angina pectoris. Silent AMI was defined as a documented characteristic electrocardiogram (ECG) or a physician's recorded diagnosis in patients with no documented history of AMI. A diagnosis of cerebrovascular disease was defined as a clearly documented history of stroke confirmed by CT or MRI, and/or a transient ischemic attack. A diagnosis of PAD was defined as the presence of at least one of the following: symptoms of intermittent lower limb claudication or ischemic pain at rest in the absence of pedal and posterior artery pulses, or bruits over the femoral arteries; previous peripheral artery surgery, endovascular interventional procedures; or lower extremity amputation due to PAD.

The erythrocyte sedimentation rate (ESR) was determined using Westergren's method (upper normal limit 30 mm/1^st^ h), and C-reactive protein (CRP) levels were measured by means of nephelometry (NA latex CRP kit, Behringwerke, Marburg, Germany; upper normal limit 5 mg/L).

### Molecular analysis of TLR-4 polymorphisms

DNA was obtained from whole blood using the standard phenol/chloroform extraction method [39]. Polymerase chain reaction (PCR) was used to amplify two small regions of the *TLR-4* gene [40]: a 248 bp fragment containing the rs4986790 Ex4+636A>G Asp299Gly polymorphism (forward primer 5′GATTAGCATACTTAGACTACTACCTCCATG3′ and reverse primer 5′GATCAACTTCTGAAAAAAGCATTCCCAC3′), and a 405 bp fragment containing the rs4986791 Ex4+936C>T Ile399Thr polymorphism (forward primer 5′GATTAGCATACTTAGACTACTACCTCCATG3′ and reverse primer 5′GATCAACTTCTGAAAAAAGCATTCCCAC3′). The two forward primers have an altered nucleotide that is useful for creating a restriction site for Ncol (the first) and Hinfl endonucleases (the second). The amplifications were performed in a 25 ul reaction mixture containing 100 uM of each dNTP, 20 pmol of each primer, and 1 unit of Taq polymerase.

The amplification profile for the 248 bp amplicon was initial denaturation at 95°C for 2 min, followed by 35 cycles (30 sec at 94°C, 30 sec at 58°C, and 30 sec at 72°C), with a final extension at 72°C for 3 min. The amplification profile for the 405 bp amplicon was initial denaturation at 95°C for 2 min, followed by 35 cycles (30 sec at 94°C, 30 sec at 66°C, and 30 sec at 72°C), with a final extension at 72°C for 3 min.

Ten microlitres of the PCR products were digested by Ncol and Hinfl restriction endonucleases, which can reveal the alleles because Ncol cuts the 248 bp amplicon only if allele A is replaced by G, and Hinfl cuts the 405 bp amplicon only if C is replaced by T. The different genotypes were revealed by means of electrophoretic analysis of the digested PCR products in 2.5% agarose gel stained with ethidium bromide (0.5 ug/mL).

### Molecular analysis of VEGF polymorphisms

DNA was extracted from the whole blood of the healthy controls and CP patients using a standard phenol, chloroform and isoamyl alcohol method [39], and *VEGF* was genotyped by PCR followed by restriction analysis.

The primers were 5′ATTTATTTTTGCTTGCCATT3′ and 5′GTCTGTCTGTCTGTCCGTCA3′ for the VEGF +634 polymorphism [31]; 5′AAGGAAGAGGAGACTCTGCG3′ and 5′TATGTGGGTGGGTGTGTCTA3′ for the VEGF +939 polymorphism [30]; and 5′CCTGGAGCGTTTTGGTTAAA3′ and 5′ATATAGGAAGCAGCTTGGAA3′ for the 18 bp I/D polymorphism [32]

PCR was carried out using a PE 9600 thermal cycler (Perkin Elmer Cetus, Norwalk, CT, USA) and a 50 mL reaction volume containing 100 ng template DNA, 50 mM KCl, 10 mM Tris-HCl, 0.1% Triton X-100, 200 mM each of dATP, dCTP, dGTP and dTTP (Amersham Pharmacia Biotech, Uppsala, Sweden), 2.5 mM MgCl2, 0.5 mM of each primer, and 1 U Taq DNA Polymerase (Perkin Elmer Cetus, Norwalk, CT, USA). Following an initial denaturation step (2 min at 95°C), the samples underwent 35 cycles consisting of 30 sec at 95°C, 30 sec at 60°C (VEGF +634) or 30 sec at 67°C (VEGF +936) or 30 sec at 55°C (18 bp I/D), and 30 sec at 72°C, followed by a final extension at 72°C for 5 min. The +634 polymorphism PCR products were digested using restriction endonuclease Bsm FI (New England Biolabs, Beverly, MA, USA), and the restriction fragments were analysed on 2% agarose gel. The 304 bp C allele remained uncut, whereas the G allele was cut into two fragments of 193 bp and 111 bp. The +936 polymorphism PCR products were digested using restriction endonuclease Nla III (New England Biolabs, Beverly, MA, USA) and the restriction fragments were analysed on 2% agarose gel. The 208 bp C allele remained uncut, whereas the T allele was cut into two fragments of 122 bp and 86 bp. The 18 bp I/D polymorphism PCR products were visualised on 2% agarose gel, which revealed two fragments: a 234 bp I allele with an 18 bp insertion, and a 216 bp D allele without insertion.

### Measurement of serum VEGF concentrations

Serum was obtained at the time of diagnosis from 17 untreated CP patients included in the genetic study, and was stored at −80°C until use. All of the patients were recruited by the Nephrology Unit of Parma University Hospital, and they all had IRF; no serum samples from patients with IAAA were available. The control group consisted of 11 age- and gender-matched healthy subjects. Serum VEGF levels were measured using the Bio-Rad Luminex assay (Bio-Rad Laboratories, Life Science Group, Hercules, CA, USA). All of the samples and standards were analysed in duplicate using a Luminex 100 for cytokine detection and Bio-Rad Manager software, version 4.0 (Bio-Rad Laboratories, Life Science Group, Hercules, CA, USA).

### Statistical analysis

The data were statistically analysed using the SPSS statistical package (SPSS Inc., Chicago, IL, USA; version 18.0, 2009). The frequencies of the alleles and genotypes in the patients and controls were compared using the chi-squared test. Fisher's exact test was used when the minimum expected value was <5. Odds ratios (ORs) were calculated together with their 95% confidence intervals (95% CI). Asymptotic look-up distribution with continuity correction was used to determine the p values. The cases and controls were tested for conformity to the Hardy-Weinberg equilibrium using a 2×2 chi-square test between observed and expected numbers [41]. The differences in serum VEGF levels were assessed using the Mann-Whitney test.

## Results

Table 1 shows the demographic and clinical characteristics of the 102 CP patients: 83 had IRF and 19 IAAA. Twenty-six (25.7%) of the 101 patients for whom relevant data were available had established atherosclerotic disease diagnosed at baseline or during follow-up. The control and case populations, and the IAAA and IRF subgroups, were tested for Hardy-Weinberg equilibrium: genotype frequencies did not reject Hardy-Weinberg equilibrium in any case.

**Table 1:** Demographic and clinical characteristics of the 102 patients with chronic periaortitis* &.

Mean age at disease onset ± SD (years)	58±12
Males/females	65.7/34.3
Idiopathic retroperitoneal fibrosis	83/102 (81.4)
Inflammatory abdominal aortic aneurysms	19/102 (18.6)
*Retroperitoneal fibrosis localisation*	
Periaortic-iliac	41/102 (40.2)
Periaortic-iliac+pericaval	53/102 (52.0)
Atypical localisation	8/102 (7.8)
Thoracic vessel or mediastinal involvement	22/58 (37.9)
Presence of atherosclerotic disease (at diagnosis or during follow-up)**	26/101 (25.7)
Systemic symptoms and/or signs§	76/100 (76.0)
Abdominal/lumbar pain	85/101 (84.2)
Constipation	29/99 (29.3)
Testicular symptoms	39/65 (60.0)
Deep vein thrombosis	16/100 (16.0)
Ureteral obstruction	77/102 (75.5)
Unilateral	28/102 (27.5)
Bilateral	50/102 (49.0)
Acute renal failure	49/102 (48.0)
Conservative treatment (stents, nephrostomies)	41/102 (40.2)
Surgical ureterolysis	28/102 (27.5)
Retroperitoneal biopsy	55/101 (54.5)
Associated autoimmune/inflammatory diseases	41/95 (43.2)
Autoimmune thyroiditis	20/88 (22.7)
ANA positivity	32/97 (33.0)
Other positive autoantibodies	46/96 (47.9)
Mean duration of follow-up ± SD (months)	42.5±38.7
Mean ESR at diagnosis ± SD (mm/hour)	68±32
Mean CRP level at diagnosis ± SD (mg/L)	41.4±43.6

* Values indicate the percentage of patients unless otherwise indicated.

& Some characteristics were not available for all 102 patients.

** Atherosclerotic disease: the presence of coronary artery disease, cerebrovascular disease or peripheral arterial disease.

§ Fever, anorexia, and weight loss.

ANA = antinuclear antibodies; ESR = erythrocyte sedimentation rate; CRP = C-reactive protein.

Table 2 shows the allele and genotype frequencies of the of *TLR-4* Asp299Gly polymorphism in the patients and controls. Eight (8.1%) of the 102 CP patients and 17 (8.5%) of the 200 controls were heterozygous for the Asp299Gly *TLR-4* allele; there were no homozygous subjects in either group. All of the the eight patients and 17 controls showed co-segregation of the Thr399Ile polymorphism, whereas none of the patients or controls had an isolated Asp299Gly polymorphism. The distribution of allele and genotype frequencies was not significantly different between the patients and controls.

**Table 2:** Alleles, genotypes and carriage rate frequencies (%) of the Toll-like receptor (TLR) 4 polymorphism Asp299Gly in patients with chronic periaortitis and control subjects*.

	Chronic periaortitis (n = 99)	Controls (n = 200)	P	OR (95% CI)
Alleles				
G	8/198 (4.0)	17/400 (4.3)	0.904	0.95 (0.40–2.24)
A	190/198 (96.0)	383/400 (95.8)		
Genotype				
G/G	0/99 (0.0)	0/200 (0.0)		
G/A	8/99 (8.1)	17/200 (8.5)	0.902	
A/A	91/99 (91.9)	183/200 (91.5)		
Carriage rate				
G/A+G/G	8/99 (8.1)	17/200 (8.5)	0.902	0.94 (0.39–2.27)
A/A	91/99 (91.9)	183/200 (91.5)		

* Values are the number/total number examined (%).

OR = odds ratio; 95% CI = 95% confidence interval.

Carriers of the G allele (G/A+G/G) were similarly represented in the two groups. Stratification of the patients by IRF or IAAA did not reveal any significant association with the *TLR-4* Asp299Gly polymorphism in either group.

We also investigated the association of the TLR-4 Asp299Gly polymorphism with CP, IRF, and IAA, after stratifying the study subjects by the presence/absence of atherosclerotic disease. There were no significant differences in allele frequencies between the patients with or without atherosclerotic disease and the healthy controls (data not shown).

The associations between the *TLR-4* Asp299Gly polymorphism and the clinical manifestations of CP shown in Table 1 were evaluated by comparing the frequencies of the clinical manifestations between the G allele carriers and non-carries; no significant differences were found (data not shown).

Table 3 shows the allele and genotype frequencies of the three *VEGF* polymorphisms in the patients and controls. There were no statistically significant differences in allele frequencies or genotype distribution. The carriers of the I allele (II or ID), −C634 allele (C634/C634 or C634/G634), and +T936 allele (T936/T936 or T936/C936) were similarly represented in the patient and control groups.

**Table 3:** Alleles, genotypes and carriage rates frequencies (%) of VEGF I/D, 936 C/T, 634 C/G in patients and control subjects*.

Variable	Chronic periaortitis (n = 102)	Controls (n = 200)	p	OR (95% CI)
Alleles				
I	92/204 (45.1)	162/400 (40.5)	0.279	1.21 (0.86–1.70)
D	112/204 (54.9)	238/400 (59.5)		
T936	24/204 (11.8)	40/400 (10.0)	0.505	1.20 (0.70–2.05)
C936	180/204 (88.2)	360/400 (90.0)		
C634	83/202 (41.1)	155/400 (38.8)	0.579	1.10 (0.78–1.56)
G634	119/202 (58.9)	245/400 (61.3)		
Genotypes				
II	24/102 (23.5)	48/200 (24.0)		
ID	44/102 (43.1)	66/200 (33.0)	0.174	
DD	34/102 (33.3)	86/200 (43.0)		
T936/T936	1/102 (1.0)	3/200 (1.5)		
T936/C936	22/102 (21.6)	34/200 (17.0)	0.596	
C936/C936	79/102 (77.5)	163/200 (81.5)		
C634/C634	12/101 (11.9)	27/200 (13.5)		
C634/G634	59/101 (58.4)	101/200 (50.5)	0.425	
G634/G634	30/101 (29.7)	72/200 (36.0)		
Carriage rates				
II+ID	68/102 (66.7)	114/200 (57.0)	0.104	1.51 (0.92–2.48)
DD	34/102 (33.3)	86/200 (43.0)		
T936/T936+T936/C936	23/102 (22.5)	37/200 (18.5)	0.404	1.28 (0.71–2.30)
C936/C936	79/102 (77.5)	163/200 (81.5)		
C634/C634+C634/G634	71/101 (70.3)	128/200 (64.0)	0.276	1.33 (0.79–2.23)
G634/G634	30/101 (29.7)	72/200 (36.0)		

* Values are the number/total number examined (%).

OR = odds ratio; 95% CI = 95% confidence intervals.

Comparing the distribution of allele frequencies of the three VEGF polymorphisms among the IAAA and IRF patients, the +936T allele was significantly more frequent in the patients with IRF (26.5% *vs* 5.3%; p = 0.046; OR 6.49 [95% CI 0.82–51.54]). We also investigated the association of the three VEGF polymorphisms with CP, IRF, and IAAA, after stratifying the patients by the presence/absence of atherosclerotic disease. There were no significant differences in allele frequencies between the patients with or without atherosclerotic disease and the healthy controls (data not shown).

The associations between the three VEGF polymorphisms and the clinical manifestations of CP as defined in Table 1 were evaluated by comparing the frequencies of the clinical manifestations between the carriers and non-carriers of the I, −C634 and +T936 alleles. The carriers of the I allele (II+ID) were significantly more likely to have ureteral obstruction (83.8% vs 58.8%, p = 006; OR 3.63 [95% CI 1.42–9.28]) and to receive conservative treatment (48.5% vs 23.5%, p = 0.015; OR 3.06 [95% CI 1.22–7.72]). The patients with deep vein thrombosis were more likely to show II allele homozygosity than those without (30.4% *vs* 11.7%; p = 0.031; OR 3.31 [95% CI 1.07–10.21] (Table 4).

**Table 4:** Carriage rate frequencies (%) of VEGF 936 C/T in patients with *IRF and **IAAA, and VEGF I/D in patients with/without ureteral obstruction, conservative treatment, and deep vein thrombosis&.

Variables	936 TC+936 TT	936 CC	P values	OR (95% CI)
IRF	22 (26.5)	61 (73.5)	0.046	6.49 [0.82–51.54]
IAAA	1 (5.3)	18 (94.7)		
	II+ID	DD		
Ureteral obstruction	57 (83.8)	20 (58.8)	0.006	3.63 [1.42–9.28]
Absence of ureteral obstruction	11 (16.2)	14 (41.2)		
	II+ID	DD		
Conservative treatment	33 (48.5)	8 (23.5)	0.015	3.06 [1.22–7.72]
No conservative treatment	35 (51.5)	26 (76.5)		
	II	DD+ID		
Deep vein thrombosis	7 (30.4)	9 (11.7)	0.031	3.31 [1.07–10.21]
Absence of deep vein thrombosis	16 (69.6)	68 (88.3)		

* Idiopathic retroperitoneal fibrosis (IRF);

** inflammatory abdominal aortic aneurysms (IAAA);

& Values are the number/total number examined (%).

OR = odds ratio; 95% CI = 95% confidence intervals.

**Figure 1:**
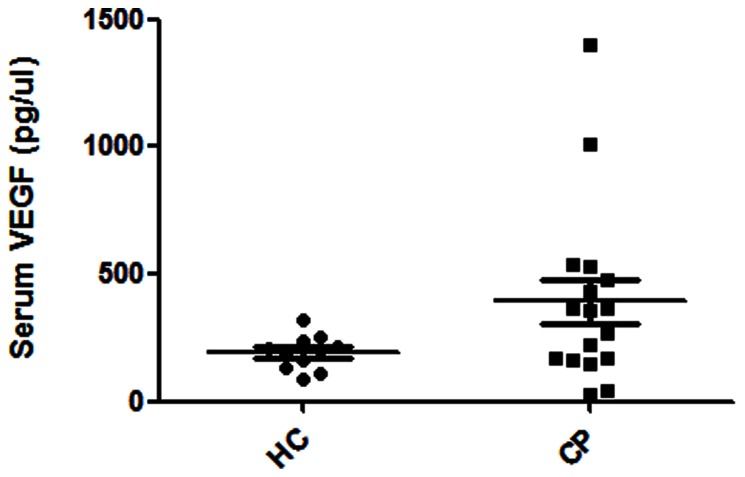
Serum VEGF levels in 17 patients with chronic periaortitis (CP) and 11 age- and gender-matched healthy controls (HC). **VEGF tended to be higher in the CP group, but the difference was not statistically significant (p = 0.081).**

Serum VEGF levels tended to be higher in the CP (IRF) patients (median 393 pg/mL, range 26–1398 pg/mL) than in the healthy controls (median 193 pg/mL, range 89–254 pg/mL), but this difference was not statistically significant (P = 0.081) (Fig. 1).

Serum VEGF levels tended to be higher in the patients with the wild-type +936 C/C genotype (median 454 pg/mL, range 44–1398 pg/mL) than in those with the C/T genotype (median 194 pg/mL, range 26–363 pg/mL, p = 0.157), and were significantly higher than the levels in the healthy controls (p = 0.037), which were similar to those observed in the patients with the C/T genotype (p = 0.948).

## Discussion

We investigated whether polymorphisms of the *TLR-4* and *VEGF* genes might be linked to susceptibility to, and the clinical manifestations of CP. TLRs play a role in many diseases, including those considered to be autoimmune in nature [10], [11]. In particular, the inappropriate activation of TLR pathways by endogenous or exogenous ligands may lead to the initiation and/or perpetuation of autoimmune responses [10], [11].

There is evidence that CP originates as a primary aortitis leading to a fibro-inflammatory reaction in the adjacent periaortic retroperitoneum. Histopathological findings demonstrate that most of the thickening of the aortic wall in CP patients is due to an enlargement of the adventitia caused by a marked inflammatory reaction. Nodular aggregates of inflammatory cells are usually centred around the adventitial vasa vasorum, which may show signs of vasculitis with fibrinoid necrosis [7]. Similarly, a subset of patients with giant cell arteritis (GCA) are characterised by perivasculitis, with the inflammation being restricted to the vasa vasorum, the periadventitial small vessels, or both [42]. Deng *et al.* [12] have elegantly demonstrated that the ligands of TLR4 can induce the activation and maturation of adventitial DCs in GCA patients, and that this initiates the cascade of events that leads to the recruitment and activation of T cells in the arterial adventitia and subsequent vessel wall inflammation. The same authors also showed that TLR4 and TLR5 induce distinct types of vasculitis in GCA patients, with TLR4 ligands causing transmural panarteritis and TLR5 ligands promoting adventitial perivasculitis [12]. It can also be postulated that TLR4 or TLR5 may also play a key role in CP. In particular, the inappropriate activation of TLR by atherosclerotic plaque antigens or other endogenous or exogenous ligands may activate DCs and trigger the marked adventitial inflammatory reaction typical of CP.

In 2002, Arbour *et al.* [14] described two co-segregating polymorphisms of the human TLR4 gene, Asp299Gly and Thr399Ile. These two SNPs are characterised by an A/G transition causing an aspartic acid/glycine substitution at amino acid location Asp299Gly (rs4986790), and a C/T transition causing a threonine/isoleucine switch at amino acid location Thr399Ile (rs4986791). Both polymorphisms occur with an allelic frequency of approximately 3–6% in Caucasians [18]. The allelic frequency in our control population (4.3%) was similar to that reported in a previous Italian study (7.2%) [43]. Furthermore, and in line with the very rare occurrence of homozygous mutations, we found no homozygous patients or controls.

Arbour *et al.* [14] were the first to investigate the functional consequence of TLR4 polymorphisms, and found that subjects with the Asp299Gly and/or Thr399Ile polymorphism had a blunted response to inhaled LPS. Furthermore, in comparison with wild-type TLR4, transfected cells with TLR4 polymorphisms showed decreased NF-κB activity leading to reduced cytokine production.

Since their identification, a series of studies have examined the impact of these polymorphisms on the incidence and course of inflammatory disorders, including vasculitides, with conflicting results [15]–[27]. The first Spanish study showed that the frequency of the TLR4 +896 G allele was significantly higher in patients with biopsy-proven GCA than in controls [23], whereas a second Spanish study [24] and an Italian [22] study did not confirm this association. In an attempt to provide a more definitive conclusion, a cumulative meta-analysis of the association of the TLR4 +896 A/G polymorphism with GCA susceptibility was made (which included these three studies), and failed to find any association [25].

Ours is the first study to examine TLR4 polymorphisms in CP patients. Our findings in a large and ethnically homogeneous Italian cohort indicate that these polymorphisms are not associated with CP susceptibility or its clinical manifestations, which suggests that they do not play an important role in the pathogenesis of CP.

Because of its angiogenic and pro-inflammatory activities, VEGF may play a role in the inflammatory process involving the aortic vasa vasorum and periaortic retroperitoneal vessels that leads to the development of CP. It is capable of upregulating endothelial adhesion molecules and chemokine expression, and Ramshaw and Parums [29] have shown that the expression of intercellular adhesion molecule-1 (ICAM-1) and vascular cell adhesion molecule-1 (VCAM-1) closely correlate with the adventitial mononuclear infiltrate in CP patients. Mangieri *et al.* [44] have recently observed that serum levels of eotaxin/chemokine (C-C motif) ligand 11 (CCL11) are significantly higher in CP patients than in healthy controls, and that it is also highly expressed by mononuclear cells in the inflammatory infiltrate of retroperitoneal biopsies obtained from CP patients. Similarly, Boiardi *et al.* [5] found that the CCR5 Δ32 polymorphism was associated with an increased risk of developing the aneurysmal form of CP, particularly in patients without established atherosclerotic disease All of these data suggest that adhesion molecules, chemokines, may play a key role in the initiation and progression of the chronic inflammatory response associated with CP.

VEGF production may be partially subject to genetic control [30]–[32]. All of the three VEGF polymorphisms evaluated in our study have been linked to variations in plasma VEGF levels and VEGF production by mononuclear cells [30]–[32]. Brenner *et al.* showed that plasma VEGF levels were significantly lower in healthy carriers of the 936T allele that in non-carriers [30], and we found similar results in our patients with IRF. The serum levels of VEGF were higher in the IRF patients with the CC genotype than in those with the C/T genotype or healthy controls, but were similar in the healthy controls and the patients with the C/T genotype. There was no association between the three polymorphisms and CP, but the +936T allele was significantly more frequent in patients with IRF than in those with IAAA. The functional relevance of this association is still unclear; we measured VEGF levels in IRF patients and healthy controls (no serum samples were available from IAAA patients), and found no significant difference between them. However, as the small number of patients might have limited our ability to detect a difference, larger studies are needed to address this issue. VEGF may be relevant to the pathogenesis of both aneurysmal and non-aneurysmal forms of CP because of its potential pro-fibrotic effects [45], [46] and its over-expression in human abdominal aortic aneurysms [47].

We found that carriers of the I allele (II+ID) were more susceptible to the development of ureteral obstruction and more likely to receive conservative treatment (i.e. stent placement). Ureteral obstruction is the most frequent complication of CP, and involves both ureters in 50–80% of cases [48]–[50].

The I/D polymorphism seems to be functionally relevant. We have shown that LPS-stimulated VEGF production by peripheral blood mononuclear cells was higher in II homozygotes than in DD homozygotes [33], [34]. By facilitating retroperitoneal mass growth and the fibrotic process, the angiogenic and pro-inflammatory properties of VEGF may be responsible for this association [45], [46]. A genetic variation in VEGF production may therefore influence susceptibility to ureteral obstruction and the need for stent placement. Interestingly, a number of studies have shown associations between the same VEGF polymorphisms encoding higher VEGF production and end-stage renal disease [51] and acute renal allograft rejection [52].

We also found that deep vein thrombosis was significantly more frequent in the II homozygous patients. It is not entirely clear why increased VEGF production may increase the risk of thrombosis. CP-related thrombosis can be due to the compression of adjacent veins by the retroperitoneal mass [50], and so higher levels of VEGF production may increase the risk of thrombosis by favouring retroperitoneal mass growth. Alternatively, higher VEGF levels could facilitate the development of thrombosis by activating endothelial cells and promoting the adhesion and activation of platelets [53]. In this regard, it is worth noting that high serum VEGF levels have been found in patients with polycythemia vera and thrombotic complications [54]. These hypotheses are not mutually exclusive.

The results of this study should be interpreted very cautiously because the small sample sizes after sub-grouping can easily induce a type 1 error. Further studies are required to replicate our findings in other populations and clarify the role of VEGF in the inflammatory events leading to vascular injury or thrombosis in patients with CP.
